# Consistency in Geometry Among Coronary Atherosclerotic Plaques Extracted From Computed Tomography Angiography

**DOI:** 10.3389/fphys.2021.715265

**Published:** 2021-10-12

**Authors:** Haipeng Liu, Aleksandra Wingert, Xinhong Wang, Jucheng Zhang, Jianzhong Sun, Fei Chen, Syed Ghufran Khalid, Yinglan Gong, Ling Xia, Jun Jiang, Jian'an Wang, Dingchang Zheng

**Affiliations:** ^1^Research Centre for Intelligent Healthcare, Coventry University, Coventry, United Kingdom; ^2^Faculty of Health, Education, Medicine, and Social Care, Anglia Ruskin University, Chelmsford, United Kingdom; ^3^Department of Radiology, The Second Affiliated Hospital, School of Medicine, Zhejiang University, Hangzhou, China; ^4^Department of Clinical Engineering, School of Medicine, The Second Affiliated Hospital, Zhejiang University, Hangzhou, China; ^5^Department of Electrical and Electronic Engineering, Southern University of Science and Technology, Shenzhen, China; ^6^Department of Medical Physics, Guy's and St Thomas' NHS Foundation Trust, London, United Kingdom; ^7^Key Laboratory for Biomedical Engineering of Ministry of Education, Institute of Biomedical Engineering, Zhejiang University, Hangzhou, China; ^8^Department of Cardiology, School of Medicine, The Second Affiliated Hospital, Zhejiang University, Hangzhou, China

**Keywords:** coronary artery disease, atherosclerotic plaques, computed tomography (CT), three-dimensional reconstruction, plaque morphology

## Abstract

**Background:** The three-dimensional (3D) geometry of coronary atherosclerotic plaques is associated with plaque growth and the occurrence of coronary artery disease. However, there is a lack of studies on the 3D geometric properties of coronary plaques. We aim to investigate if coronary plaques of different sizes are consistent in geometric properties.

**Methods:** Nineteen cases with symptomatic stenosis caused by atherosclerotic plaques in the left coronary artery were included. Based on attenuation values on computed tomography angiography images, coronary atherosclerotic plaques and calcifications were identified, 3D reconstructed, and manually revised. Multidimensional geometric parameters were measured on the 3D models of plaques and calcifications. Linear and non-linear (i.e., power function) fittings were used to investigate the relationship between multidimensional geometric parameters (length, surface area, volume, etc.). Pearson correlation coefficient (*r*), *R*-squared, and *p*-values were used to evaluate the significance of the relationship. The analysis was performed based on cases and plaques, respectively. Significant linear relationship was defined as *R*-squared > 0.25 and *p* < 0.05.

**Results:** In total, 49 atherosclerotic plaques and 56 calcifications were extracted. In the case-based analysis, significant linear relationships were found between number of plaques and number of calcifications (*r* = 0.650, *p* = 0.003) as well as total volume of plaques (*r* = 0.538, *p* = 0.018), between number of calcifications and total volume of plaques (*r* = 0.703, *p* = 0.001) as well as total volume of calcification (*r* = 0.646, *p* = 0.003), and between the total volumes of plaques and calcifications (*r* = 0.872, *p* < 0.001). In plaque-based analysis, the power function showed higher R-squared values than the linear function in fitting the relationships of multidimensional geometric parameters. Two presumptions of plaque geometry in different growth stages were proposed with simplified geometric models developed. In the proposed models, the exponents in the power functions of geometric parameters were in accordance with the fitted values.

**Conclusion:** In patients with coronary artery disease, coronary plaques and calcifications are positively related in number and volume. Different coronary plaques are consistent in the relationship between geometry parameters in different dimensions.

## Introduction

Coronary artery disease is a cardiovascular disease that has been the leading cause of death globally (Malakar et al., 2019). The formation of atherosclerotic plaques in the coronary arteries plays a key role in the development of coronary artery disease (CAD). With the growth of an atherosclerotic plaque, the stenosis in the affected coronary artery reduces the myocardial blood flow and oxygen supply. Evidence has shown that the geometry of a plaque, which determines the severity of arterial stenosis, is related to the risks of clinical events, including myocardial infarction in patients with CAD (Choi et al., 2015; Lee et al., 2019a). Further investigation of coronary plaque geometry is, therefore, recognised as an important pathway toward understanding the pathophysiology and improving the diagnosis and treatment of CAD (Ratiu et al., 2018).

Currently, computerised tomography (CT) scan is the most commonly used medical imaging technique in the diagnosis of CAD (Gaur et al., 2016). The coronary computed tomography angiography (CCTA) images have a high spatial resolution to reflect the geometry and morphology of coronary plaques (Stefanini Giulio and Windecker, 2015). CCTA can achieve a higher resolution than cardiac magnetic resonance imaging (MRI) (Liu et al., 2021). The severity of luminal stenosis evaluated by CCTA was in similar accuracy as that obtained from intravascular ultrasound (IVUS) (Lee et al., 2019b). The coronary angiography (CAG) is also widely used in the diagnosis of CAD. Compared with MRI, IVUS, and CAG, CCTA is low-cost, non-invasive, and safe to use on patients with implants (Wang et al., 2018). CCTA can visualise different types of plaque composition. Non-calcified, partially calcified, and calcified plaques could be extracted separately based on their attenuation values (Daghem et al., 2020). Therefore, CCTA has been widely applied in the analysis of coronary plaque geometry (Rinehart et al., 2011; Ratiu et al., 2019).

Using the three-dimensional reconstruction of plaque geometry from CCTA images, many geometric parameters can be accurately estimated, including the burden (i.e., the severity of stenosis), size, diameter, and composition of coronary artery plaques (Liu et al., 2021). The geometry of a plaque can influence the hemodynamic parameters, such as wall shear stress, which, in turn, influence the evolution of plaque geometry (Yamamoto et al., 2017; Arzani, 2020; Pleouras et al., 2020a). For example, the clinical observation and computational simulation have disclosed that plaques grow faster in the downstream areas than the upstream areas (Arzani, 2020). Considering the haemodynamics-driven plaque growth, in this study, we hypothesize that different coronary plaques may be consistent in geometry.

The geometric consistency among different objects can be investigated using the relationships between multidimensional geometric parameters (i.e., parameters defined in different geometric dimensions). For example, sphericity has been proposed to evaluate the geometric consistency between different objects. It was defined as the ratio of the surface area of a sphere, which has the same volume as the given particle to the surface area of that particle (Li et al., 2012). The surface-area–volume ratio has been applied in investigating the taxonomic groups of insects (Kühsel et al., 2017), as well as the relationship between human brain size and cortex folding (Toro et al., 2008). However, despite the fact the many geometric parameters have been applied in the evaluation of coronary plaques (Liu et al., 2021), as far as we know, the geometric consistency of coronary plaques has not been comprehensively investigated using multidimensional geometric parameters based on patient-specific CCTA imaging data.

In order to comprehensively understand coronary plaque geometry and its evolution, we aim to investigate the relationship between different multidimensional geometric parameters of coronary plaques reconstructed from CCTA images. Considering the geometric and hemodynamic differences between left and right coronary arteries, we focus on the plaques in left coronary arteries in this pilot study.

## Methods

### Patients and CCTA Imaging Protocol

In this retrospective study, the CCTA imaging data were collected from 25 patients with CAD in the Second Affiliated Hospital of Zhejiang University in China from 2015 to 2016 with approval from the local ethics committee for sharing and analysing retrospective anonymised patient data with informed consent form waived. Details of the CCTA scan protocol are listed in Table 1. Each scan was visually examined for visible coronary stenosis, culprit lesions, and calcifications. The resultant datasets of systolic and diastolic scans were compared regarding the quality of images, with the better one selected for analysis.

**Table 1:** CCTA data acquisition.

**Parameter name**	**Name/qty**
Scanner type	Somatom definition flash
Number of slices	128
Patient's position	Supine
Patient's heart rate (beats per minute, mean ± SD)	70.125 ± 14.047
Contrast type	Ultravist 370
Any beta-blockers?	No
Any nitro-glycerine?	Yes- one subject, the rest- no
Time of the scan (minutes, mean ± SD)	2.805 ± 2.464

In six patients, there was no observable plaque or significant stenosis (>50% in diameter) in left coronary artery. In total, 19 patients (13 males, 6 females, mean ± SD of age: 63.4 ± 9.5 years) were finally included for analysis, as listed in Table 2. The observer was blinded for the case diagnosis. The quantity, location, and type of atherosclerotic plaques have been confirmed by a radiologist and a cardiologist.

**Table 2:** Characteristics of patients.

Patients, *n*	19
Female, *n*	6
Age (years, mean ± SD)	63.421 ± 9.532
Total number of plaques, *n*	49
Number of plaques per case (mean ± SD)	2.579 ± 1.387
Total number of calcifications, *n*	56
Number of calcifications per case (mean ± SD)	3.158 ± 1.803
Mean calcified plaque volume (per case, *mm*^3^, mean ± SD)	24.137 ± 24.156
Mean whole plaque volume (per case,*mm*^3^, mean mean ± SD)	151.084 ± 115.431
Mean plaque surface area (per case,*mm*^2^, mean mean ± SD)	313.995 ± 186.056

### 3D Reconstruction of Coronary Arteries

The 3D geometry of coronary arteries and plaques was reconstructed from the CCTA images using the software MIMICS 20.0 (Materialise N.V., Belgium). The reconstruction method was semiautomatic, using the Coronary Segmentation Tool of Mimics Medical Suite. Firstly, the position of the aorta was marked. Then, with start and end points marked on the CCTA image dataset, an arterial segment can be automatically reconstructed with the result saved in an independent set (a “mask” in MIMICS). For the left coronary artery tree, artery segments were extracted from the left main coronary artery (LM) to major branches, including the left anterior descending artery (LAD) and left circumflex artery (LCX), and, finally, the distal branches. The small branches (diameter <1 mm, or blurred structure) were trimmed off. The left coronary artery tree was derived by connecting artery segments using the union operation of different sets.

### Extraction of Coronary Atherosclerotic Plaques: A Two-Step Approach

**Figure 1:**
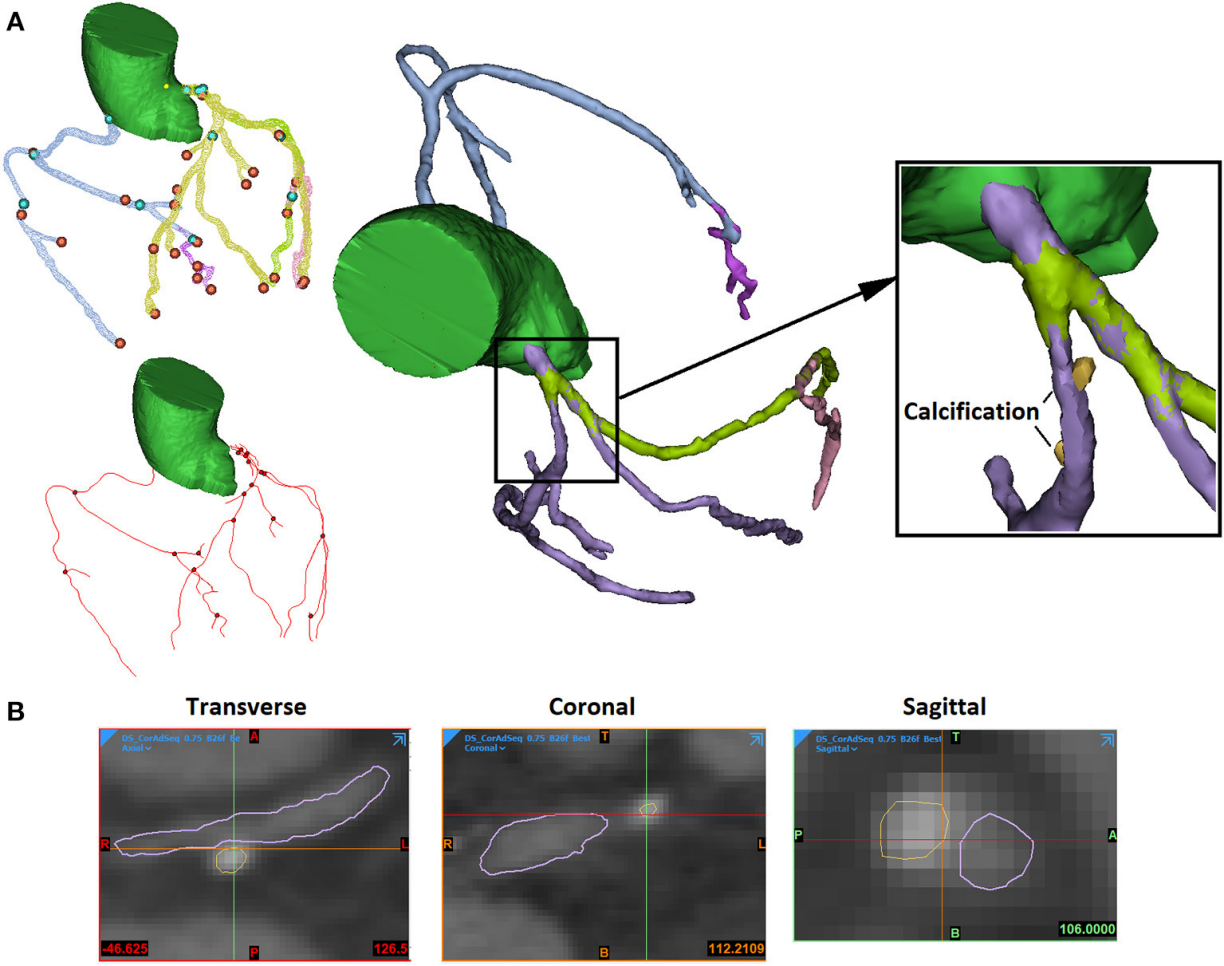
The extraction of arterial lumen and calcification. **(A)** The 3D view. The blue and orange points present the start and the end points of extracted artery segments. The red lines and black points present the centerlines of artery segments and connecting points (left). The arterial segments were extracted in different masks (in different colours) and connected (middle). The calcifications were extracted independently (right). **(B)** The 2D views of stenosed artery in three directions (sagittal, coronal, and transverse).

**Figure 2:**
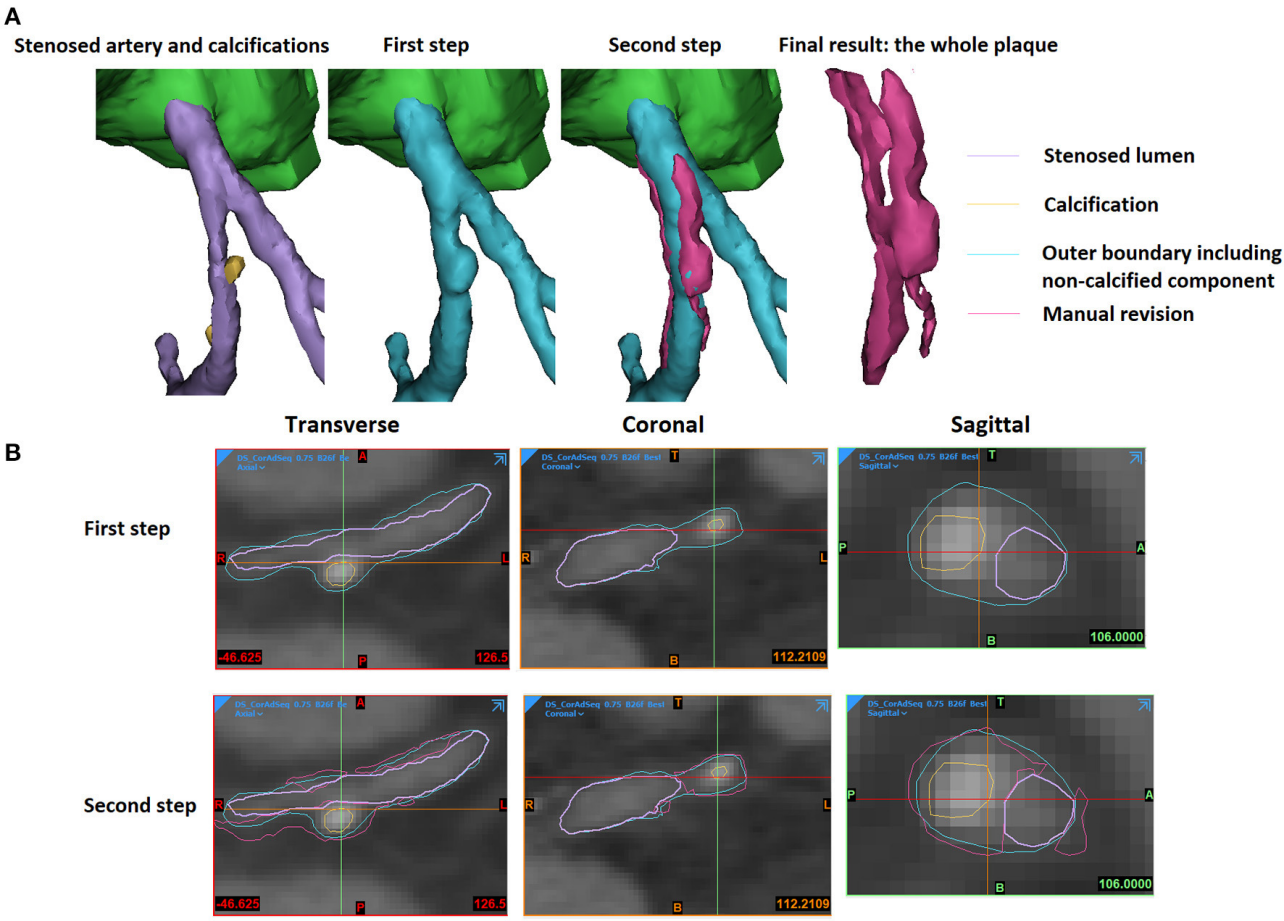
The extraction of an atherosclerotic plaque. **(A)** The 3D view. **(B)** The 2D views. The stenosed artery lumen, calcification, outer boundary that wraps vessel lumen and non-calcified components, as well as the manually revised results were shown in purple, yellow, cyan, and magenta colours in the 3D and 2D views.

The 3D reconstruction of coronary plaques was based on a semiautomatic two-step approach. It is difficult to distinguish between fibrotic, fibro-fatty, and fatty atherosclerotic lesions. Therefore, in this study, the plaque components were categorised as calcified (calcification) and non-calcified. Firstly, based on the position of plaque on the two-dimensional (2D) CCTA images, the spatial ranges of plaque were set in three directions (sagittal, coronal, and transverse). In the 3D space defined by the ranges, the plaque was automatically extracted using thresholds of CT attenuation value (in Hounsfield units, or HU). Due to the difference in plaque composition, the CT attenuation value varies among different scans. Therefore, the thresholds were set patient specifically. Low attenuation values (0–150 HU) were used to identify non-calcified plaques, whereas high attenuation values (>150–1,334 HU) were used for calcified components. Firstly, the calcifications were automatically extracted (Figure 1). The demarcation between non-calcified plaque components, vessel lumen, and other tissues was inaccurate due to the varying local attenuation. Therefore, a mask was developed by using a wide HU range for all the non-calcified components, including the vessel wall. By using union Boolean operation, an outer boundary was derived that wraps the vessel lumen and the whole plaque (the cyan part in Figure 2).

In the second step, the plaque geometry was further modified. Considering the blooming artefact caused by calcification, the surrounding non-calcified components have higher HU values. The boundaries between non-calcified components and calcification or artery lumen were checked and adjusted manually. The areas opposite to the calcification were also reviewed to add the non-calcified plaque components that were not detected by the threshold used in the first step due to the effect of surrounding tissues (the magenta part in Figure 2).

### Measurement of Multidimensional Geometric Parameters

The measurement of multidimensional geometric parameters in different dimensions was performed using the Materialise 3-Matic software (Materialise N.V., Belgium) on the whole plaques and calcifications, respectively.

**Figure 3:**
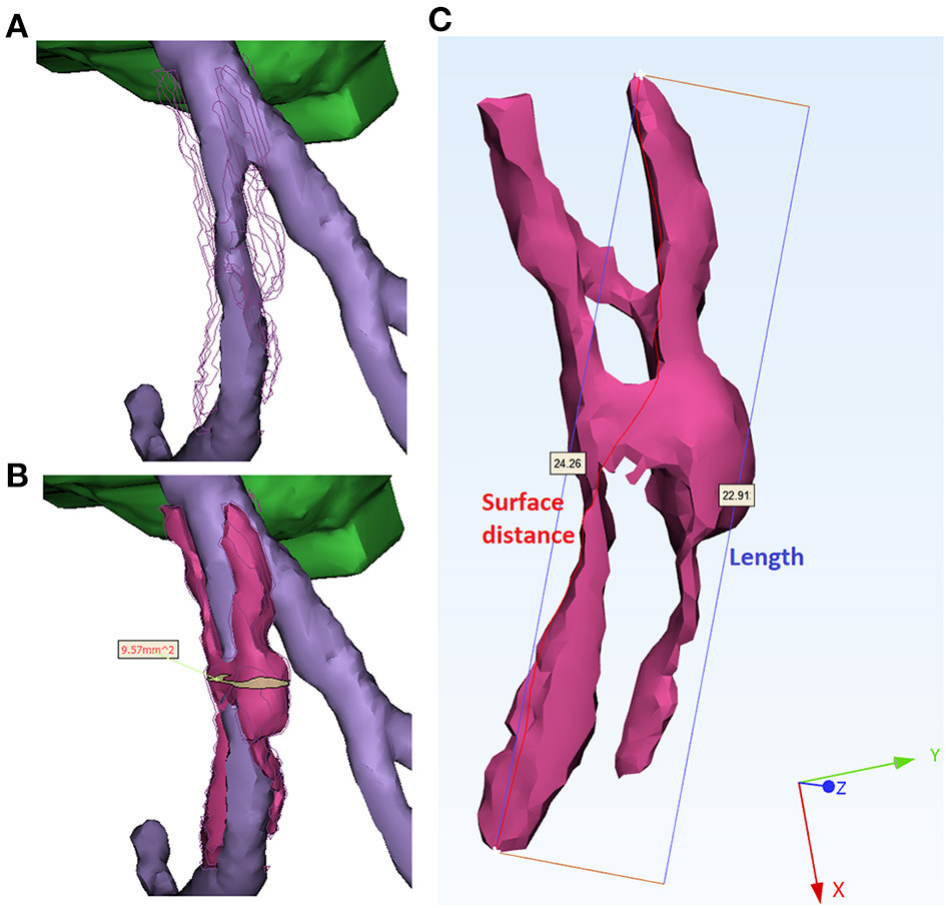
Measurement of geometric parameters. **(A)** Stenosed artery lumen. The surface of plaque was indicated by polylines for better comparison. **(B)** Measurement of the cross-section area of a plaque (magenta). The cross-section with the maximal area (yellow) was derived perpendicularly to the centerline of arterial segment (purple). **(C)** Measurement of length and surface distance of a plaque. The red curve (24.26 mm) and blue segment (22.91 mm) illustrate the shortest distance on the surface and in the 3D space, respectively.

For each plaque and calcification, firstly, the volume and the surface area were measured automatically. Secondly, the length, surface distance, cross-sectional area, and diameter were measured manually. The length of a plaque or calcification was defined as the longest straight-line distance between its proximal and distal ends. The surface distance was defined as the length of the shortest path on the surface between the proximal and distal ends. For the whole plaque, the cross-section area was measured on the cross-section perpendicular to the local centerline of the arterial segment with the maximal area (Figure 3). The cross-section diameter was measured as the longest distance on the cross-section area. Compared with the plaques, calcifications had much smaller size, rounder shape, and were positioned in variable directions in spatial distribution. Therefore, the cross-section area and the diameter were not measured on calcifications.

### Statistical Analysis

The statistical analysis was performed using SPSS 24.0 software (IBM SPSS Inc., Chicago, IL, USA) based on each case, plaque, and calcification, respectively.

In the case-based analysis, the sum of volumes of different plaques or calcifications was calculated for each subject. The relationships among the numbers and volumes of plaques and calcifications were investigated using simple linear regression with Pearson correlation coefficient (*r*), coefficient of determination (R-squared), and *p*-value calculated. A significant linear relationship was defined as *r* > 0.5 (*R*-squared > 0.25) and *p* < 0.05. A strong linear relationship was defined as *r* > 0.8 (*R*-squared > 0.64) and *p* < 0.05.

We investigated the multidimensional geometric parameters in the plaque- and calcification-based analyses. Each pair of parameters was in different dimensions except for length and surface distance which can reflect the smoothness of the plaque surface. In the plaque-based analysis, simple linear regression analysis was performed between different geometric parameters (i.e., volume, surface area, length, surface distance, cross-section area, and cross-section diameter) of plaques. Similarly, in the calcification-based analysis, simple linear regression analysis was performed on the geometric parameters measured from each calcification (i.e., volume, surface area, length, and surface distance). If any significant linear relationship was found between two parameters of different dimensions (e.g., surface area and volume), the non-linear regression analysis based on power function was performed. The results of linear and non-linear regressions were compared using their R-squared values. Finally, to further investigate the relationship between plaques and calcifications in volume, simple linear regression analysis was performed in the plaque-based analysis between the plaque volume and the total volume of calcifications in the plaque.

### Geometric Modelling

Based on the analysis of multidimensional parameters (see Results), we proposed two models to describe the geometry of small (patchy) and big (circular) coronary plaques. All the plaques were simplified as a part of a cylindrical shell with uniform thickness (**Figure 6**). To evaluate if the models can accurately reflect the plaque geometry, the ratio between plaque length, surface area, and volume was calculated and compared with the obtained measurement results.

## Results

### Summary

In the 19 subjects, in total, 49 atherosclerotic plaques and 56 calcifications were extracted from the left coronary arteries. In each case, the numbers of plaques and calcifications were (mean ± SD) 2.579 ± 1.387 and 3.158 ± 1.803; the mean calcification volume and mean whole plaque volume were 24.137 ± 24.156 *mm*^3^ and 151.084 ± 115.431 *mm*^3^, respectively. The plaques located in all the major branches of left coronary artery tree: LAD (in 18 subjects), LCX (in 7 subjects), LM (in 5 subjects), as well as the diagonal (in 4 subjects), and marginal (in 3 subjects) branches. The CA scores ranged from 1.2 to 856.4. Therefore, the plaques in this study were diverse in location and composition.

### Case-Based Analysis

The results of case-based analysis showed a strong relationship between the plaques and calcifications in number and volume. Significant linear relationships were observed between the number of plaques and the number of calcifications (*r* = 0.650, *p* = 0.003), between the number of plaques and the volume of plaques (*r* = 0.538, *p* = 0.018), between the number of calcifications and the volume of plaques (*r* = 0.703, *p* = 0.001), and between the number of calcifications and the volume of calcifications (*r* = 0.646, *p* = 0.003). The volume of plaques and volume of calcifications showed a strong linear relationship (*r* = 0.872, *r* < 0.001) (Table 3).

**Table 3:** Linear regression results of case-based analysis.

	**Number of plaques**	**Number of calcifications**	**Total volume of plaques**	**Total volume of calcification**
Number of plaques		*r =* 0.650, *p* = 0.003^*^	*r =* 0.538, *p =* 0.018^*^	*r =* 0.411, *p =* 0.080
Number of calcifications			*r =* 0.703, *p =* 0.001^*^	*r =* 0.646, *p =* 0.003^*^
Total volume of plaques				*r =* 0.872, p<0.001^**^

* *marks significant linear relationship (R-squared > 0.25 (i.e., r > 0.5) and p < 0.05)*.

** *marks strong linear relationship (R-squared > 0.64 (i.e., r > 0.8) and p <0.05)*.

### Plaque-Based and Calcification-Based Analysis: Linear and Non-linear Regressions

For the plaques, a significant linear relationship was found between the volume and the cross-section area (*r* = 0.506, *p* < 0.001). Strong linear relationships were found between the volume and the surface area (*r* = 0.944, *p* < 0.001), volume and length (*r* = 0.861, *p* < 0.001), volume and surface distance (*r* = 0.843, *p* < 0.001), surface area and length (*r* = 0.925, *p* < 0.001), surface area and surface distance (*r* = 0.908, *p* < 0.001), length and surface distance (*r* = 0.997, *p* < 0.001), as well as cross-section diameter and cross-section area (r = 0.866, *p* < 0.001). There was a significant linear relationship between the plaque volume and the total volume of calcifications in each plaque (*r* = 0.754, *p* < 0.001).

**Figure 4:**
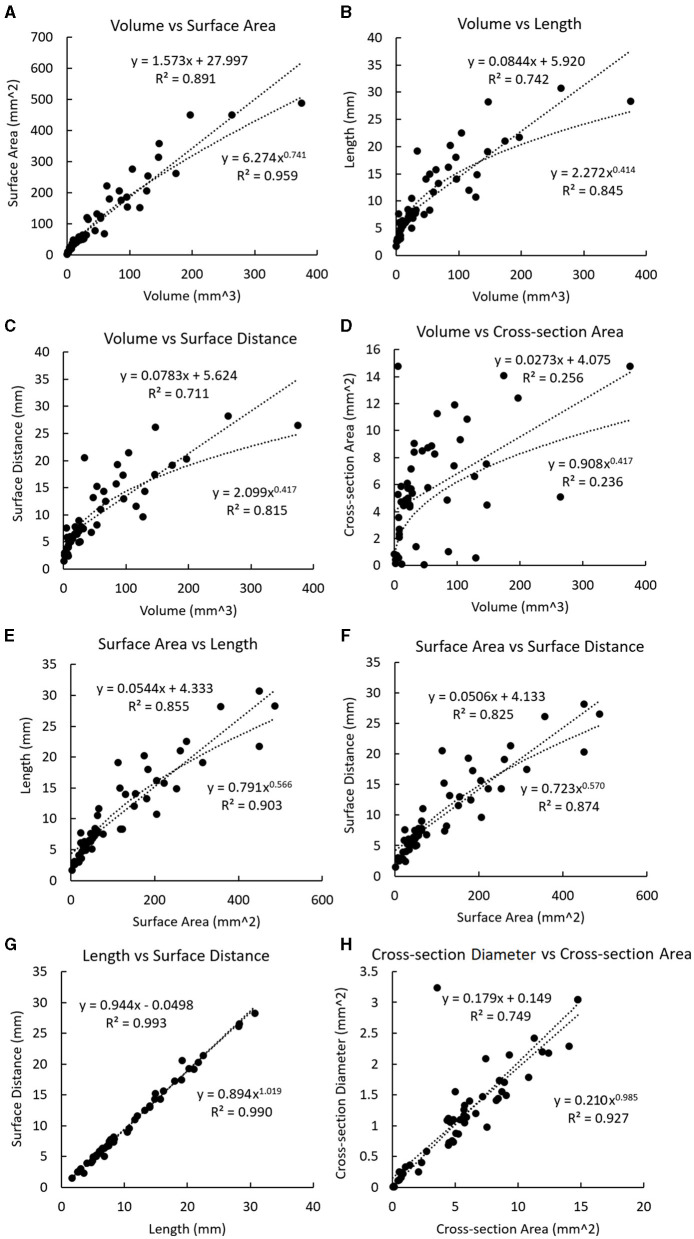
Linear and non-linear regression fittings on the geometric parameters of plaques. The regression line and curve are shown in dotted lines. **(A)** The volume and the surface area. **(B)** Volume and length. **(C)** Volume and surface distance. **(D)** Volume and the cross-section area. **(E)** The surface area and length. **(F)** The surface area and surface distance. **(G)** Length and surface distance. **(H)** Cross-section diameter and the cross-section area.

In Figure 4, the non-linear regression shows a higher R-squared value than linear regression in most relationships except between the volume and the cross-section area where the relationship is just above the significant threshold (*R*-squared: 0.256 and 0.236 for linear and non-linear regressions), as well as between the length and the surface distance where the values are very approximate (*R*-squared: 0.993 and 0.990 for linear and nonlinear regressions).

**Figure 5:**
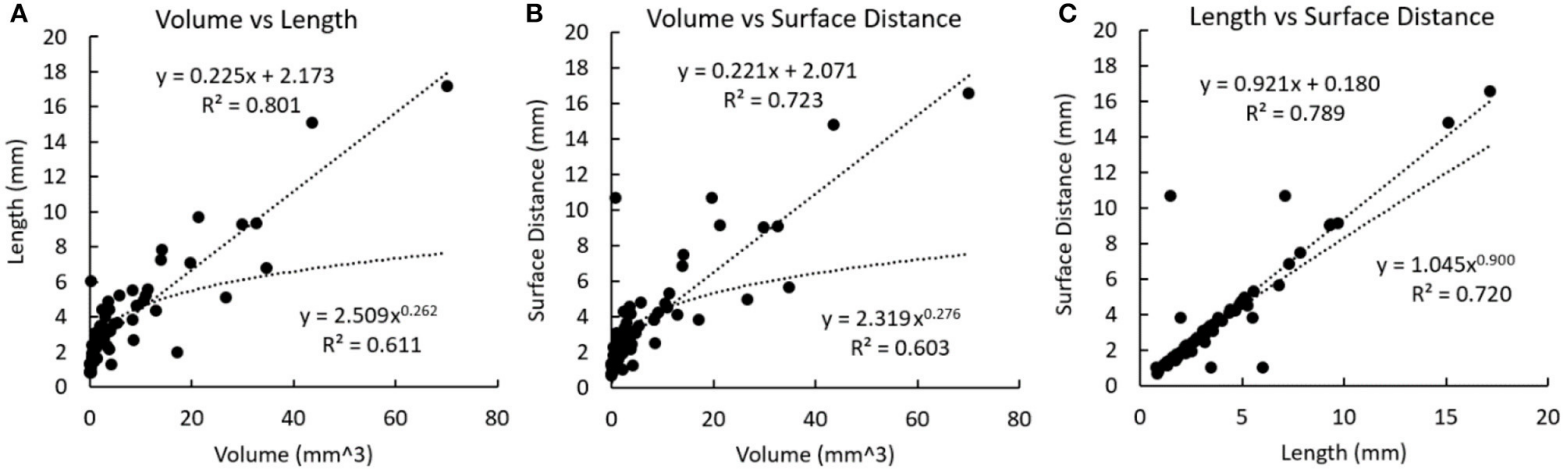
Linear and non-linear regression fittings on the geometric parameters of calcifications. The regression line and curve are shown in dotted lines. **(A)** Volume and length. **(B)** Volume and surface distance. **(C)** Length and surface distance.

In the calcification-based analysis, strong linear relationships were found between volume and length (*r* = 0.895, *p* < 0.001), volume and surface distance (*r* = 0.850, *p* < 0.001), as well as length and surface distance (*r* = 0.888, *p* < 0.001). In Figure 5, the linear regression always shows a higher *R*-squared value than the non-linear regression. All the relationships in Figure 5 are weaker than the corresponding ones in Figure 4.

### Geometric Modelling of Coronary Plaques: Two Presumptions

#### Presumption 1: Cylindrical Ring Shape of Fully Developed Plaques

**Figure 6:**
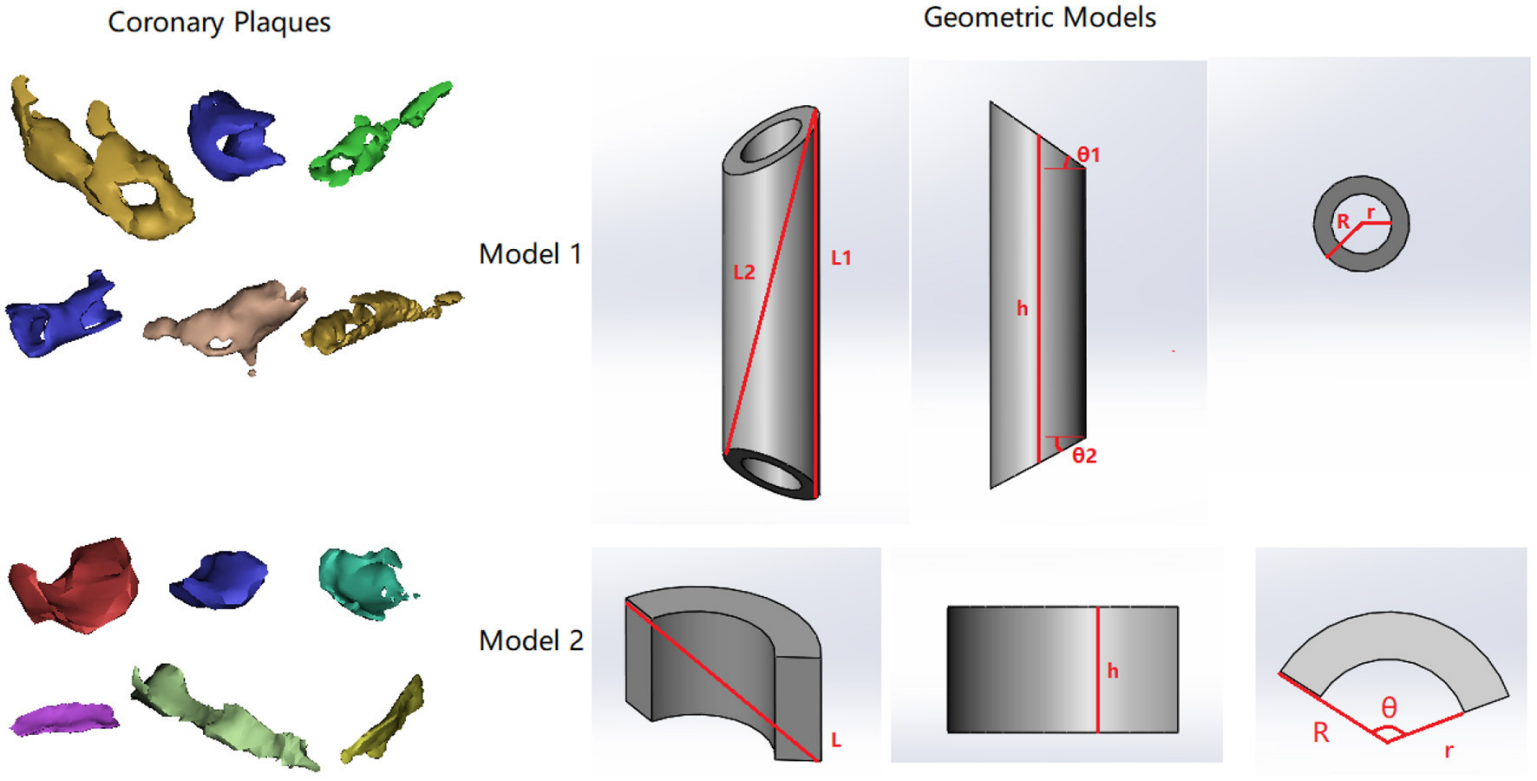
Two types of coronary atherosclerotic plaques and corresponding geometric models. In both models, R, r, and h are the external radius, internal radius, and height on the centerline. In model 1, L_1_ describes the longest segment along the longitudinal direction on the surface. L_2_ describes the segment from one end of L_1_ to the opposite side. L_1_ and L_2_ form a plane across the centerline of the cylinder. θ_1_ and θ_2_ describe the dihedral angles between slanted surface planes and the perpendicular cross-section plane at both ends. In model 2, θ describes the circumferential range of the partial ring.

It can be observed in Figure 6 that the fully developed plaques are nearly symmetric circumferentially but asymmetric along the artery wall. In a large-scale study of coronary plaque geometry using intravascular ultrasound, 50.2% were concentric in 1,441 cross-sections (Komiyama et al., 2016). Longitudinally, atherosclerotic plaques often grow faster along one side of the artery wall due to the difference in hemodynamic effect (Gnasso et al., 1997; Samady et al., 2011). Therefore, we presume that a fully developed plaque has a nearly cylindrical ring shape in the middle segment and extends slantly toward the proximal and distal arterial segments. Accordingly, we developed a model of cylindrical ring shape with slanted cross-sections at both ends (Model 1 in Figure 6). The ratio between the surface area (S) and the volume (V) can be derived as


(1) $$\begin{array}{l} \frac{S}{V}=\frac{2}{R-r}+\frac{1}{h}\left(\frac{1}{\cos\theta_{1}}+\frac{1}{\cos\theta_{2}}\right) \end{array}$$

where R, r, and h are the external radius, internal radius, and height on the centerline. θ_1_ and θ_2_ describe the dihedral angles between the surface planes and the perpendicular cross-section plane at both ends. When the size of plaque increases, the value of $\frac{S}{V}$ decreases: $\frac{S}{V}=0$, which is in accordance with the observation in Figure 4A. The exponent in the power function relationship between S and V can be derived as


(2) $$\begin{array}{l} \frac{lnS}{lnV}=\frac{ln\pi +ln\left(R+r\right)+ln\left[2h+\left(R-r\right)\left(\frac{1}{\cos\theta_{1}}+\frac{1}{\cos\theta_{2}}\right)\right]}{ln\pi +lnh+ln\left(R+r\right)+ln\left(R-r\right)} \end{array}$$

which can be estimated based on the following two hypotheses on plaque geometry evolution:

***Hypothesis 1.1:*** during the growth of a plaque, the values of R, r, and h are linearly related while θ_1_ and θ_2_ are independent. Under this hypothesis, it can be derived that
  (3) $$\begin{array}{l} \underset{V\to \infty }{\lim}\frac{lnS}{lnV}=\frac{2}{3} \end{array}$$
***Hypothesis 1.2:*** with the growth of plaque, the atherosclerosis extends asymmetrically on the artery wall toward the proximal and distal arterial segments, where the values of R, r, $\frac{1}{\cos\theta_{1}}$, $\frac{1}{\cos\theta_{2}}$, and h are linearly related. It can be derived that
  (4) $$\begin{array}{l} \underset{V\to \infty }{\lim}\frac{lnS}{lnV}=1 \end{array}$$

In Figure 4A, the exponent in the power function fitting is 0.741, which is between $\frac{2}{3}$ and 1. Therefore, this geometric model reflects the relationship between the surface area and the volume in real coronary plaques.

To investigate the relationship between plaque length and volume, the length was calculated under two hypotheses:

***Hypothesis 1.3:*** the plaque length (L) locates along the external wall
  (5) $$\begin{array}{l} \text{L }=\text{ h }+\text{ R}\left(\text{tan}\theta_{1}\;+\text{ tan}\theta_{2}\right) \end{array}$$
***Hypothesis 1.4:*** the plaque length (L) goes across the geometry
  (6) $$\begin{array}{l} L=\sqrt{{\left(2R\right)}^{2}+{\left(h+R\tan\theta_{1}-R\tan\theta_{2}\right)}^{2}} \end{array}$$

In both cases, it can be derived that $\frac{lnL}{lnV}\approx \frac{1}{3}$ under hypothesis 1.1, and $\frac{lnL}{lnV}\approx \frac{2}{3}$ under hypothesis 1.2. In Figure 4B, the exponent in the power function fitting is 0.414, which is between $\frac{1}{3}$ and $\frac{2}{3}$. Therefore, this model is in accordance with the geometric properties of real coronary plaques.

#### Presumption 2: Partial Cylindrical Ring Shape of Early-Stage Plaques

In the early stage of plaque formation, the vessel wall thickening often emerges from one side of coronary artery and extends both longitudinally and circumferentially. Therefore, we proposed a model that describes the geometry of an early-stage plaque that covers circumstantially a part of the artery wall (Model 2 in Figure 6, in which θ describes the circular range of the plaque and θ < π). The ratio between the surface area and the volume can be derived as:


(7) $$\begin{array}{l} \frac{S}{V}=2\left[\frac{2}{\theta \left(R+r\right)}+\frac{1}{h}+\frac{1}{R-r}\right] \end{array}$$

When the size of a plaque increases, the value of $\frac{S}{V}$ decreases: $\underset{V\to \infty }{\lim}\frac{S}{V}=0$, which is in accordance with the observation in Figure 4A. The exponent in the power function relationship between S and V can be calculated as


(8) $$\begin{array}{l} \frac{lnS}{lnV}=\frac{ln\left[2h\left(R-r\right)+\theta \left(R+h\right)\left(R-r+h\right)\right]}{ln\theta +lnh+ln\left(R+r\right)+ln\left(R-r\right)-ln2} \end{array}$$

We investigated the plaque geometry under the following hypothesis:

***Hypothesis 2.1:*** The growth of atherosclerotic plaque extends in axial and circumferential directions, where h, R, and r are linearly related, while θ increases toward 2π.

Thus, the following relationships could be derived:


(9) $$\begin{array}{l} \underset{V\to \infty }{\lim}\frac{lnS}{lnV}=\frac{2}{3} \end{array}$$

(10) $$\begin{array}{l} L=\sqrt{h^{2}+{\left(2R\sin\frac{\theta }{2}\right)}^{2}} \end{array}$$

(11) $$\begin{array}{l} \frac{L}{V}=\frac{2\sqrt{h^{2}+{\left(2R\sin\frac{\theta }{2}\right)}^{2}}}{\theta h\left(R^{2}-r^{2}\right)} \end{array}$$

(12) $$\begin{array}{l} \frac{lnL}{lnV}=\frac{ln2+0.5ln\left(h^{2}+{\left(2R\sin\frac{\theta }{2}\right)}^{2}\right)}{ln\theta +lnh+ln\left(R+r\right)+ln\left(R-r\right)} \end{array}$$

(13) $$\begin{array}{l} \underset{V\to \infty }{\lim}\frac{lnL}{lnV}=\frac{1}{3} \end{array}$$

In Figure 4, the values of exponents for $\underset{V\to \infty }{\lim}\frac{lnS}{lnV}$ and $\underset{V\to \infty }{\lim}\frac{lnL}{lnV}$ are 0.741 and 0.414, which are in accordance with the values derived from the model (difference <0.08 and <0.09 for $\frac{S}{V}$ and $\frac{L}{V},$ respectively). Therefore, this model is also in accordance with the geometric properties of real coronary plaques.

## Discussion

The results disclosed the geometric consistency among coronary atherosclerotic plaques of different sizes. Based on the results, we proposed two simplified geometric models of coronary plaques whose geometric properties were in accordance with the measurement results. As far as we know, this is the first study on the geometry properties of coronary plaques in different dimensions.

In this pilot study, the coronary plaques were extracted from CCTA images and manually revised. As aforementioned, some invasive imaging methods, such as IVUS and optical coherence tomography, can achieve a high accuracy in detecting the geometry and morphology of atherosclerotic plaques while CCTA is non-invasive and widely used in the diagnosis of CAD. Additionally, CCTA has high reproducibility (Symons et al., 2016) and reliability in evaluating coronary plaques (Dweck et al., 2016). However, automatic extraction of coronary plaques from CCTA images could cause under- or over-estimation of plaque size, especially for calcified plaques where blooming artefact could increase the attenuation value of adjacent tissues, leading to the inaccurate estimation of plaque volume (Liu et al., 2021). Additionally, the automatic separation of a non-calcified (lipid or fibrotic) plaque from surrounding tissues could be difficult due to the similarity in attenuation value (Liu et al., 2019). Therefore, for CCTA images, the manual revision could reduce the inaccuracy in plaque geometry. These results were also assessed and corrected by experienced radiologists to provide a basis for reliable geometric analysis.

In some studies on the computational simulation of CAD, simplified 2D models of different atherosclerotic plaques have been developed, including carotid plaque models (Yuan et al., 2015; Shahidian and Hassankiadeh, 2017) and idealised plaque models (Shimizu and Ohta, 2015). On the cross-section plane perpendicular to the centerline of a local arterial segment, the circular, elliptical, crescent, and irregular shapes have been used to describe the plaque geometry where the internal and external boundaries of the arterial wall were modelled using a circle and an ellipse, respectively (Shahidian and Hassankiadeh, 2017). By changing the size of the crescent shape, Kumar and Balakrishnan investigated the stress on coronary plaques with different sizes (Krishna Kumar and Balakrishnan, 2005). On the cross-section plane along the artery centerline, Konala et al. used a simplified trapezoid model to describe the longitudinal coronary plaque geometry along the local artery segment (Konala et al., 2011). As a result, the 3D geometry of the plaque had adjustable slopes of the proximal and distal segments (Karimi et al., 2012, 2014c). Some smoother shapes were also developed to describe the longitudinal plaque geometry. Yasutomoa and Makotoa used a semicircle model to investigate the recirculation area distal to the plaque (Shimizu and Ohta, 2015). To describe the longitudinal change of radius along the stenotic artery segment in a more accurate way, Moreno and Bhaganagar used a cosine function to describe the boundary between coronary plaque and the lumen in the longitudinal direction (Moreno and Bhaganagar, 2013). With these smoother functions, saddle-shaped 3D plaque geometry could be derived to describe the geometry of eccentric plaques (Karimi et al., 2014a,b; Yuan et al., 2015). However, the geometric models in existing studies were highly simplified without validation using patient-specific imaging data. Based on the patient-specific CCTA imaging data, we found the consistency in geometry among different coronary plaques and developed two geometric models whose geometry properties were in accordance with the measured results. These results provide an important reference for the pathophysiological and computational studies on coronary plaques.

The power function relationship between multidimensional geometric parameters (volume, surface area, and length) of coronary plaques is a novel finding. As we observed, coronary plaques often grow along the artery wall, forming curved shells, rings, or more complicated shapes, which are quite different from a sphere (Figure 6). Therefore, instead of using sphericity to evaluate geometric consistency of coronary plaques, we directly investigated the relationship between geometric parameters in different dimensions. The consistency of plaque geometry is related to plaque growth. With a certain degree of stenosis, the plaque geometry has a direct effect on the turbulence flow characteristics in the surrounding areas (Bhaganagar, 2009). As a result, an area with low values of wall shear stress (WSS), which is a hemodynamic parameter directly related to endothelial function and atherosclerosis, will appear in the distal segment of the plaque, accelerating the plaque growth (Liu et al., 2018; Arzani, 2020). In a study on 900 artery segments extracted from 94 patient-specific coronary arteries, Pleouras et al. found that the plaque growth and degree of stenosis predicted using the baseline WSS values derived from computational simulation were in accordance with the follow-up clinical observation (Pleouras et al., 2020b). Therefore, the plaque geometry can influence the plaque growth by hemodynamic effects in pathological mechanisms, which contributes to the geometric consistency of different coronary plaques.

On the other hand, geometrical parameters of coronary plaques can provide information about many CAD-related clinical risks: the severity of ischemia, the risky areas of plaque growth, and the stability of arteriosclerotic plaques (Choi et al., 2015; Driessen Roel et al., 2018). Therefore, the accurate evaluation of plaque geometry in different dimensions can help to determine the pathological and morphological properties of coronary plaques which will improve the diagnosis, treatment, and management of CAD.

The calcifications and plaques showed different geometric properties. Firstly, the case-based analysis disclosed that plaques and calcifications were significantly correlated in number and total volume (Table 3). This is in accordance with the fact that calcification occurs in the mature plaques. The plaque-based analysis showed a significant relationship between plaque volume and total calcification volume in the plaque. In carotid and coronary arteries, the volume of calcification is closely related to the intima-media thickness (IMT) (Araki et al., 2015). Pathologically, calcification often appears in the later stages of the atherosclerotic lesion (Shioi and Ikari, 2017). Thus, in the case-based analysis, the total calcification volume was not significantly related to the total number of plaques (Table 3). Secondly, compared with the plaques, the calcifications had less significant relationships between multidimensional geometric parameters (Figures 4, 5), which suggested that the geometry of calcification is more irregular. In atherosclerotic plaques, the formation of calcification is influenced by different pathophysiological mechanisms, including the death of inflammatory cells, releasing of matrix vesicles, reduced local expression of mineralisation inhibitors, and induction of bone formation resulting from differentiation of pericytes and/or vascular smooth muscle cells (Nakahara et al., 2017). Therefore, the distribution of calcifications is discontinuous with irregular geometry. It was observed that calcification itself exists in a diverse range of morphologies from spherical microcalcifications to large irregular-shaped macrocalcifications (Cahalane and Walsh, 2021). Additionally, the calcification geometry extracted from CCTA images is influenced by the image quality and the blooming artefact (Liu et al., 2021). Due to the limited resolution of imaging techniques, detailed shape features of calcifications have not been analysed (Shi et al., 2020a). The shape of calcification was found to be significantly related to the rupture of carotid plaques where the calcifications in the ruptured plaques displayed a remarkably lower ratio between the long axis and the short axis (Shi et al., 2020b). Therefore, the 3D geometry of coronary calcification may provide valuable information to improve the diagnosis and prognosis of CAD, which deserves further investigation.

This study has some limitations. Firstly, only 19 subjects were included in this pilot study, in which there was no plaque rupture. All the plaques were from the left coronary arteries. The size and composition of plaques vary across different epicardial coronary arteries (Bax et al., 2021) and are influenced by some physiological factors, such as age and gender (Khosa et al., 2013). The plaque diversity deserves further consideration. Secondly, the plaques were manually extracted, which is time-consuming, unstandardized, and difficult for large-scale clinical application. Finally, we focused on the geometry of the plaques. The relationship between plaque geometry and other pathophysiological factors, including local coronary artery geometry and medical history of CAD, deserves further investigation. In future studies, the large-scale study based on automatic extraction algorithms of plaque and calcification could be considered to cover different types of coronary plaques and subjects. The geometry of calcification could be further investigated using more accurate imaging technologies (e.g., IVUS). Based on the large-scale data, the relationship between the plaque geometry and the pathophysiological factors of CAD could be investigated.

## Conclusion

The coronary plaques with different sizes showed consistency in geometry with power function relationships between geometric parameters of different dimensions. The calcifications and plaques are significantly related in terms of number and total volume.

## Data Availability Statement

The original contributions presented in the study are included in the article/supplementary material, further inquiries can be directed to the corresponding author/s.

## Ethics Statement

The studies involving human participants were reviewed and approved by Ethics Committee of the Second Affiliated Hospital of Zhejiang University School of Medicine. Written informed consent for participation was not required for this study in accordance with the national legislation and the institutional requirements.

## Author Contributions

HL conceived and planned the study and proposed the geometry models. AW reconstructed the models of coronary artery and plaques and measured the geometric parameters. HL, AW, and XW checked the original images and manually revised the models. HL and AW performed the statistical analysis. DZ supervised the project that led to production of the results. All authors contributed to the discussion and revision of the manuscript and concur with the current submitted version.

## Funding

This study was supported by the National Natural Science Foundation of China (Grant No. 61828104), the Zhejiang Provincial Department of Science and Technology (Grant No. 2020C03016), and the Basic Research Foundation of Shenzhen (Grant No. GJHZ20180928155002157).

## Conflict of Interest

The authors declare that the research was conducted in the absence of any commercial or financial relationships that could be construed as a potential conflict of interest.

## Publisher's Note

All claims expressed in this article are solely those of the authors and do not necessarily represent those of their affiliated organizations, or those of the publisher, the editors and the reviewers. Any product that may be evaluated in this article, or claim that may be made by its manufacturer, is not guaranteed or endorsed by the publisher.

## References

[B1] Araki T. Ikeda N. Dey N. Acharjee S. Molinari F. Saba L.. (2015). Shape-based approach for coronary calcium lesion volume measurement on intravascular ultrasound imaging and its association with carotid intima-media thickness. J. Ultrasound Med. 34, 469–482. 10.7863/ultra.34.3.469 25715368

[B2] Arzani A. (2020). Coronary artery plaque growth: A two-way coupled shear stress–driven model. Int. J. Numer. Method. Biomed. Eng. 36:e3293. 10.1002/cnm.3293 31820589

[B3] Bax A. M. Van Rosendael A. R. Ma X. Van Den Hoogen I. J. Gianni U. Tantawy S. W.. (2021). Comparative differences in the atherosclerotic disease burden between the epicardial coronary arteries: quantitative plaque analysis on coronary computed tomography angiography. Europ. Heart J. 22, 322–330. 10.1093/ehjci/jeaa275 33215192

[B4] Bhaganagar K. (2009). Direct numerical simulation of flow in stenotic channel to understand the effect of stenotic morphology on turbulence. J. Turbulence 10:N41. 10.1080/14685240903468796

[B5] Cahalane R. M. Walsh M. T. (2021). Nanoindentation of calcified and non-calcified components of atherosclerotic tissues. Exp. Mech. 61, 67–80. 10.1007/s11340-020-00635-z

[B6] Choi G. Lee J. M. Kim H.-J. Park J.-B. Sankaran S. Otake H.. (2015). Coronary artery axial plaque stress and its relationship with lesion geometry: application of computational fluid dynamics to coronary CT angiography. JACC Cardiovasc. Imaging 8, 1156–1166. 10.1016/j.jcmg.2015.04.024 26363834

[B7] Daghem M. Bing R. Fayad Zahi A. Dweck Marc R. (2020). Noninvasive imaging to assess atherosclerotic plaque composition and disease activity. JACC Cardiovasc. Imaging 13, 1055–1068. 10.1016/j.jcmg.2019.03.033 31422147 PMC10661368

[B8] Driessen Roel S. Stuijfzand Wijnand J. Raijmakers Pieter G. Danad I. Min James K. Leipsic Jonathon A.. (2018). Effect of plaque burden and morphology on myocardial blood flow and fractional flow reserve. J. Am. Coll. Cardiol. 71, 499–509. 10.1016/j.jacc.2017.11.054 29406855

[B9] Dweck M. R. Doris M. K. Motwani M. Adamson P. D. Slomka P. Dey D.. (2016). Imaging of coronary atherosclerosis — evolution towards new treatment strategies. Nat. Rev. Cardiol. 13, 533–548. 10.1038/nrcardio.2016.79 27226154

[B10] Gaur S. Øvrehus K. A. Dey D. Leipsic J. Bøtker H. E. Jensen J. M.. (2016). Coronary plaque quantification and fractional flow reserve by coronary computed tomography angiography identify ischaemia-causing lesions. Eur. Heart J. 37, 1220–1227. 10.1093/eurheartj/ehv690 26763790 PMC4830909

[B11] Gnasso A. Irace C. Carallo C. De Franceschi M. S. Motti C. Mattioli P. L.. (1997). *In vivo* association between low wall shear stress and plaque in subjects with asymmetrical carotid atherosclerosis. Stroke 28, 993–998. 10.1161/01.STR.28.5.993 9158640

[B12] Karimi A. Navidbakhsh M. Faghihi S. Shojaei A. Hassani K. (2012). A finite element investigation on plaque vulnerability in realistic healthy and atherosclerotic human coronary arteries. Proc. Inst. Mech. Eng. H 227, 148–161. 10.1177/0954411912461239 23513986

[B13] Karimi A. Navidbakhsh M. Razaghi R. (2014a). A finite element study of balloon expandable stent for plaque and arterial wall vulnerability assessment. J. Appl. Phys. 116:044701. 10.1063/1.4891019

[B14] Karimi A. Navidbakhsh M. Razaghi R. Haghpanahi M. (2014b). A computational fluid-structure interaction model for plaque vulnerability assessment in atherosclerotic human coronary arteries. J. Appl. Phys. 115:144702. 10.1063/1.4870945

[B15] Karimi A. Navidbakhsh M. Shojaei A. Hassani K. Faghihi S. (2014c). Study of plaque vulnerability in coronary artery using mooney–rivlin model: a combination of finite element and experimental method. Biomed. Eng. 26:1450013. 10.4015/S1016237214500136

[B16] Khosa F. Khan A. N. Nasir K. Bedayat A. Malik Z. Jon A. F.. (2013). Comparison of coronary plaque subtypes in male and female patients using 320-row MDCTA. Atherosclerosis 226, 428–432. 10.1016/j.atherosclerosis.2012.11.033 23287639 PMC4758671

[B17] Komiyama H. Takano H. Nakamura S. Takano M. Hata N. Yasushi M.. (2016). Geographical predisposition influences on the distribution and tissue characterisation of eccentric coronary plaques in non-branching coronary arteries: cross-sectional study of coronary plaques analysed by intravascular ultrasound. Cardiovasc. Ultrasound 14:47. 10.1186/s12947-016-0090-3 27876049 PMC5120430

[B18] Konala B. C. Das A. Banerjee R. K. (2011). Influence of arterial wall-stenosis compliance on the coronary diagnostic parameters. J. Biomech. 44, 842–847. 10.1016/j.jbiomech.2010.12.011 21215971

[B19] Krishna Kumar R. Balakrishnan K. R. (2005). Influence of lumen shape and vessel geometry on plaque stresses: possible role in the increased vulnerability of a remodelled vessel and the “shoulder” of a plaque. Heart 91:1459. 10.1136/hrt.2004.049072 15774611 PMC1769160

[B20] Kühsel S. Brückner A. Schmelzle S. Heethoff M. Blüthgen N. (2017). Surface area–volume ratios in insects. Insect Sci. 24, 829–841. 10.1111/1744-7917.12362 27234132

[B21] Lee J. M Choi Ki H. Koo B.-K. Park J. Kim J. Hwang D.. (2019a). Prognostic implications of plaque characteristics and stenosis severity in patients with coronary artery disease. J. Am. Coll. Cardiol. 73, 2413–2424. 10.1016/j.jacc.2019.02.060 31097161

[B22] Lee S.-E. Villines T. C. Chang H.-J. (2019b). Should CT replace IVUS for evaluation of CAD in large-scale clinical trials: effects of medical therapy on atherosclerotic plaque. J. Cardiovasc. Comput. Tomogr. 13, 248–253. 10.1016/j.jcct.2019.06.017 31351840

[B23] Li T. Li S. Zhao J. Lu P. Meng L. (2012). Sphericities of non-spherical objects. Particuology 10, 97–104. 10.1016/j.partic.2011.07.005

[B24] Liu H. Gong Y. Leng X. Xia L. Wong K. S. Ou S.. (2018). Estimating current and long-term risks of coronary artery *in silico* by fractional flow reserve, wall shear stress and low-density lipoprotein filtration rate. Biomed. Phys. Eng. Express 4:025006. 10.1088/2057-1976/aa9a09

[B25] Liu H. Wingert A. Jian'an Wang J. Z. Wang X. Sun J. Chen F.. (2021). Extraction of coronary atherosclerotic plaques from computed tomography imaging: a review of recent methods. Front. Cardiovasc. Med. 8:597568. 10.3389/fcvm.2021.597568 33644127 PMC7903898

[B26] Liu J. Jin C. Feng J. Du Y. Lu J. Zhou J. (2019). “A vessel-focused 3D convolutional network for automatic segmentation and classification of coronary artery plaques in cardiac CTA,” in Statistical Atlases and Computational Models of the Heart. Atrial Segmentation and LV Quantification Challenges, eds. Pop M. Sermesant M. Zhao J. Li S. Mcleod K. Young A.. (Granada: Springer International Publishing), 131–141. 10.1007/978-3-030-12029-0_15

[B27] Malakar A. K. Choudhury D. Halder B. Paul P. Uddin A. Chakraborty S. (2019). A review on coronary artery disease, its risk factors, and therapeutics. J. Cell. Physiol. 234, 16812–16823. 10.1002/jcp.28350 30790284

[B28] Moreno C. Bhaganagar K. (2013). Modeling of stenotic coronary artery and implications of plaque morphology on blood flow. Model. Simulat. Eng. 2013:390213. 10.1155/2013/390213

[B29] Nakahara T. Dweck Marc R. Narula N. Pisapia D. Narula J. Strauss H. W. (2017). Coronary artery calcification: from mechanism to molecular imaging. JACC Cardiovasc. Imaging 10, 582–593. 10.1016/j.jcmg.2017.03.005 28473100

[B30] Pleouras D. S. Sakellarios A. I. Loukas V. S. Kyriakidis S. Fotiadis D. I. (2020a). “Prediction of the development of coronary atherosclerotic plaques using computational modeling in 3D reconstructed coronary arteries,” in 2020 42nd Annual International Conference of the IEEE Engineering in Medicine & Biology Society (EMBC) (Montreal, QC: IEEE), 2808–2811. 10.1109/EMBC44109.2020.9176219 33018590

[B31] Pleouras D. S. Sakellarios A. I. Tsompou P. Kigka V. Kyriakidis S. Rocchiccioli S.. (2020b). Simulation of atherosclerotic plaque growth using computational biomechanics and patient-specific data. Sci. Rep. 10:17409. 10.1038/s41598-020-74583-y 33060746 PMC7562914

[B32] Ratiu M. Chitu M. Benedek I. Benedek T. Kovacs I. Rat N.. (2018). Impact of coronary plaque geometry on plaque vulnerability and its association with the risk of future cardiovascular events in patients with chest pain undergoing coronary computed tomographic angiography—the GEOMETRY study: Protocol for a prospective clinical trial. Medicine 97:e13498. 10.1097/MD.0000000000013498 30544446 PMC6310548

[B33] Ratiu M. Rat N. Nyulas T. Moldovan G. Rus V. Benedek T.. (2019). Coronary plaque geometry and thoracic fat distribution in patients with acute chest pain–a CT angiography study. J. Cardiovasc. Emerg. 5, 18–24. 10.2478/jce-2019-0001

[B34] Rinehart S. Vazquez G. Qian Z. Murrieta L. Christian K. Voros S. (2011). Quantitative measurements of coronary arterial stenosis, plaque geometry, and composition are highly reproducible with a standardized coronary arterial computed tomographic approach in high-quality CT datasets. J. Cardiovasc. Comput. Tomogr. 5, 35–43. 10.1016/j.jcct.2010.09.006 21131252

[B35] Samady H. Eshtehardi P. Mcdaniel M. C. Suo J. Dhawan S. S. Maynard C.. (2011). Coronary artery wall shear stress is associated with progression and transformation of atherosclerotic plaque and arterial remodeling in patients with coronary artery disease. Circulation 124, 779–788. 10.1161/CIRCULATIONAHA.111.021824 21788584

[B36] Shahidian A. Hassankiadeh A. G. (2017). Stress analysis of internal carotid artery with low stenosis level: the effect of material model and plaque geometry. J. Mech. Med. Biol. 17:1750098. 10.1142/S0219519417500981

[B37] Shi X. Gao J. Lv Q. Cai H. Wang F. Ye R.. (2020a). Calcification in Atherosclerotic Plaque Vulnerability: Friend or Foe? Front. Physiol. 11:56. 10.3389/fphys.2020.00056 32116766 PMC7013039

[B38] Shi X. Han Y. Li M. Yin Q. Liu R. Wang F.. (2020b). Superficial calcification with rotund shape is associated with carotid plaque rupture: an optical coherence tomography study. Front. Neurol. 11:563334. 10.3389/fneur.2020.563334 33071946 PMC7530839

[B39] Shimizu Y. Ohta M. (2015). Influence of plaque stiffness on deformation and blood flow patterns in models of stenosis. Biorheology 52, 171–182. 10.3233/BIR-14016 26406780

[B40] Shioi A. Ikari Y. (2017). Plaque calcification during atherosclerosis progression and regression. J. Atheroscler. Thromb. 25, 294–303. 10.5551/jat.RV17020 29238011 PMC5906181

[B41] Stefanini Giulio G. Windecker S. (2015). Can coronary computed tomography angiography replace invasive angiography? Circulation 131, 418–426. 10.1161/CIRCULATIONAHA.114.008148 25623124

[B42] Symons R. Morris J. Z. Wu C. O. Pourmorteza A. Ahlman M. A. Lima J.. (2016). Coronary CT angiography: variability of ct scanners and readers in measurement of plaque volume. Radiology 281, 737–748. 10.1148/radiol.2016161670 27636027 PMC5131836

[B43] Toro R. Perron M. Pike B. Richer L. Veillette S. Pausova Z.. (2008). Brain size and folding of the human cerebral cortex. Cerebral Cortex 18, 2352–2357. 10.1093/cercor/bhm261 18267953

[B44] Wang Y. Osborne M. T. Tung B. Li M. Li Y. (2018). Imaging cardiovascular *Calcification* 7:e008564. 10.1161/JAHA.118.008564 29954746 PMC6064897

[B45] Yamamoto E. Siasos G. Zaromytidou M. Coskun Ahmet U. Xing L. Bryniarski K.. (2017). Low endothelial shear stress predicts evolution to high-risk coronary plaque phenotype in the future. Circulation 10:e005455. 10.1161/CIRCINTERVENTIONS.117.005455 28768758

[B46] Yuan J. Teng Z. Feng J. Zhang Y. Brown A. J. Gillard J. H.. (2015). Influence of material property variability on the mechanical behaviour of carotid atherosclerotic plaques: a 3D fluid-structure interaction analysis. Int. J. Numer. Method. Biomed. Eng. 31:e02722. 10.1002/cnm.2722 25940741 PMC4528233

